# Nesfatin-1_30−59_ Injected Intracerebroventricularly Differentially Affects Food Intake Microstructure in Rats Under Normal Weight and Diet-Induced Obese Conditions

**DOI:** 10.3389/fnins.2015.00422

**Published:** 2015-11-23

**Authors:** Philip Prinz, Pauline Teuffel, Vanessa Lembke, Peter Kobelt, Miriam Goebel-Stengel, Tobias Hofmann, Matthias Rose, Burghard F. Klapp, Andreas Stengel

**Affiliations:** ^1^Division of General Internal and Psychosomatic Medicine, Charité Center for Internal Medicine and Dermatology, Charité-UniversitätsmedizinBerlin, Germany; ^2^Department of Internal Medicine and Institute of Neurogastroenterology, Martin-Luther-KrankenhausBerlin, Germany

**Keywords:** anorexigenic, brain-gut-axis, NUCB2, satiation, satiety

## Abstract

Nesfatin-1 is well-established to induce an anorexigenic effect. Recently, nesfatin-1_30−59_, was identified as active core of full length nesfatin-1_1−82_ in mice, while its role in rats remains unclear. Therefore, we investigated the effects of nesfatin-1_30−59_ injected intracerebroventricularly (icv) on the food intake microstructure in rats. To assess whether the effect was also mediated peripherally we injected nesfatin-1_30−59_ intraperitoneally (ip). Since obesity affects the signaling of various food intake-regulatory peptides we investigated the effects of nesfatin-1_30−59_ under conditions of diet-induced obesity (DIO). Male Sprague–Dawley rats fed *ad libitum* with standard diet were icv cannulated and injected with vehicle (5 μl ddH_2_O) or nesfatin-1_30−59_ at 0.37, 1.1, and 3.3 μg (0.1, 0.3, 0.9 nmol/rat) and the food intake microstructure assessed using a food intake monitoring system. Next, naïve rats were injected ip with vehicle (300 μl saline) or nesfatin-1_30−59_ (8.1, 24.3, 72.9 nmol/kg). Lastly, rats were fed a high fat diet for 10 weeks and those developing DIO were icv cannulated. Nesfatin-1 (0.9 nmol/rat) or vehicle (5 μl ddH_2_O) was injected icv and the food intake microstructure assessed. In rats fed standard diet, nesfatin-1_30−59_ caused a dose-dependent reduction of dark phase food intake reaching significance at 0.9 nmol/rat in the period of 4–8 h post injection (−29%) with the strongest reduction during the fifth hour (−75%), an effect detectable for 24 h (−12%, *p* < 0.05 vs. vehicle). The anorexigenic effect of nesfatin-1_30−59_ was due to a reduction in meal size (−44%, *p* < 0.05), while meal frequency was not altered compared to vehicle. In contrast to icv injection, nesfatin-1_30−59_ injected ip in up to 30-fold higher doses did not alter food intake. In DIO rats fed high fat diet, nesfatin-1_30−59_ injected icv reduced food intake in the third hour post injection (−71%), an effect due to a reduced meal frequency (−27%, *p* < 0.05), while meal size was not altered. Taken together, nesfatin-1_30−59_ is the active core of nesfatin-1_1−82_ and acts centrally to reduce food intake in rats. The anorexigenic effect depends on the metabolic condition with increased satiation (reduction in meal size) under normal weight conditions, while in DIO rats satiety (reduction in meal frequency) is induced.

## Introduction

Nesfatin-1_1−82_ was discovered in 2006 by Mori et al. as an anorexigenic peptide derived from the rat brain (Oh-I et al., 2006). It is post-translationally processed from the gene encoding nucleobindin2 (NUCB2) by the pro-hormone-convertase 1/3 (Oh-I et al., 2006). NUCB2 consists of a 24 amino acid N-terminal signal peptide and a protein structure containing 396 amino acids, but only the 396 amino acid sequence is cleaved into the N-terminal nesfatin-1_1−82_, nesfatin-2_85−163_, and the C-terminal nesfatin-3_166−396_ (Oh-I et al., 2006). It was shown that nesfatin-1_1−82_, but not nesfatin-2_85−163_ or nesfatin-3_166−396_, reduces food intake and body weight gain in rats after injection into the third brain ventricle (Oh-I et al., 2006). The anorexigenic effect of central nesfatin-1_1−82_ injected into the lateral, third and fourth brain ventricle, into the cisterna magna or directly into the lateral hypothalamic area, the paraventricular nucleus, or the dorsal vagal complex has been confirmed by various independent groups in several studies in rats (Yosten and Samson, 2009, 2010; Chen et al., 2012; Könczöl et al., 2012; Xia et al., 2012; Dong et al., 2014), mice (Atsuchi et al., 2010; Goebel et al., 2011), and goldfish (Gonzalez et al., 2010; Kerbel and Unniappan, 2012). This converging evidence points toward a physiological role of nesfatin-1_1−82_ in the regulation of food intake.

Following these initial studies, the anorexigenic effect of nesfatin-1 was further characterized investigating the underlying food intake microstructure providing deeper insight into the mechanisms underlying the reduction of food intake (Geary, 2005). In mice, nesfatin-1_1−82_ injected intracerebroventricularly (icv) reduced food intake with a delayed onset, an effect due to a reduction in meal size and meal frequency (Goebel et al., 2011) indicating a stimulatory effect on satiation and satiety (Strubbe and Woods, 2004). However, as species differences often play a role, this characterization is pending in rats.

Recently, the active core of nesfatin-1_1−82_ has been identified, namely the mid fragment nesfatin-1_30−59_ (Shimizu et al., 2009), while the N-terminal nesfatin-1_1−29_, and the C-terminal nesfatin-1_60−82_ had no anorexigenic effect in mice (Shimizu et al., 2009; Stengel et al., 2012a). Although it is not clear yet whether this processing occurs *in vivo*, the characterization is an important step toward a better understanding of the ligand-receptor interaction, keeping in mind that the nesfatin-1 receptor remains to be identified. Again, the characterization of the anorexigenic effect of nesfatin-1_30−59_ is lacking for rats.

After the initial identification of NUCB2/nesfatin-1 in the brain, NUCB2 mRNA, and NUCB2/nesfatin-1 protein expression was also detected in the periphery (Stengel et al., 2009b). Interestingly, the major source of NUCB2/nesfatin-1 is the stomach with much higher NUCB2 mRNA expression levels compared to the brain (Stengel et al., 2009b) likely to act as a gut-brain peptide, a hypothesis supported by the observation that nesfatin-1 is able to cross the blood-brain barrier (Pan et al., 2007). Interestingly, on a cellular level NUCB2/nesfatin-1 is co-expressed with ghrelin, the major orexigenic peptide signaling from the gut to the brain (Al Massadi et al., 2014), within the same cells in rats (Stengel et al., 2009b), and humans (Stengel et al., 2013a). This led to the assumption that these endocrine cells in the stomach can regulate food intake in both directions, either stimulate *via* the release of ghrelin or inhibit *via* the release of NUCB2/nesfatin-1 (Stengel and Taché, 2012b). However, data on the peripheral effects of nesfatin-1 on food intake are inconsistent. While several studies observed no effects of nesfatin-1 on food intake in rats (Stengel et al., 2009a) or mice (Goebel et al., 2011), one study described an anorexigenic effect of nesfatin-1_30−59_ following intraperitoneal (ip) injection of high doses in mice (Shimizu et al., 2009). However, no data in rats exist so far.

The therapeutic potential of NUCB2/nesfatin-1 was discussed in several review articles (Stengel et al., 2013b), especially in the light of its leptin-independent signaling pathway (Oh-I et al., 2006), a hormone known to be less active under conditions of obesity (also known as leptin resistance) (Crujeiras et al., 2015). However, a detailed characterization of the food intake microstructure under conditions of diet-induced obesity (DIO), a well-established animal model for alimentary obesity (Lutz and Woods, 2012) frequently found in humans, has not been performed yet.

Therefore, the aims of the current study were to investigate the effect of icv injected nesfatin-1_30−59_ on the food intake microstructure in normal weight rats. Next, we investigated whether the effect could also be observed following ip injection in rats. Lastly, to characterize the effect of nesfatin-1_30−59_ on the food intake microstructure in obese animals, DIO rats fed a high fat diet were generated and nesfatin-1_30−59_ injected icv.

## Materials and methods

### Animals

Male Sprague–Dawley rats (Harlan-Winkelmann Co., Borchen, Germany) weighing 280–350 g were first group housed (4 rats/group) under controlled illumination (06.00–18.00 h) and temperature (21–23°C). During this time rats were handled daily to become accustomed to the interaction with the investigators (daily control of body weight, light hand restraint for subsequent icv, or ip injections). Rats had *ad libitum* access to standard rodent diet (D12450B, Research Diets, Inc., Jules Lane, New Brunswick, NJ, USA) and tap water. Animal care and experimental procedures followed institutional ethics guidelines, conformed to the requirements and were approved by the state authority for animal research conduct (Landesamt für Gesundheit und Soziales Berlin, LaGeSo Berlin; animal protocol # G 0131/11).

### Peptides

Rat nesfatin-1_30−59_ (Bachem AG, Weil am Rhein, Germany) was aliquoted in sterile distilled water and stored at –80°C until further use. Purity was assessed by HPLC and mass spectroscopy (manufacturer's information). Directly before administration, rat nesfatin-1_30−59_ was further diluted in sterile ddH_2_O for icv injection or in sterile 0.9% saline (B. Braun AG, Melsungen, Germany) for ip injection to reach the final concentrations detailed below.

### Diets

For the induction of DIO, rats were fed a high fat diet (D12451, 45% calories from fat, 35% from carbohydrates, and 20% from protein, 4.7 kcal/g diet, Research Diets Inc.) for a period of 10 weeks. Control rats were kept on a standard rodent diet (D12450B, 10% calories from fat, 70% from carbohydrates, and 20% from protein, 3.9 kcal/g diet, Research Diets Inc.). Body weight and food intake were assessed daily. At the end of the 10-week feeding period, the 50% of rats gaining the most body weight were selected as DIO.

### Intracerebroventricular cannulation

Rats were chronically icv cannulated as described before (Stengel et al., 2010a,b). Briefly, rats were anesthetized with an ip injection of 100 mg/kg ketamine (Ketanest™, Curamed, Karlsruhe, Germany) and 10 mg/kg xylazine (Rompun™, 2%, Bayer, Leverkusen, Germany). Afterwards, rats were placed in a stereotactic apparatus to implant a chronic 22-gauge guide cannula into the right lateral brain ventricle. The coordinates for the placement (from bregma: 0.8 mm posterior, 1.5 mm right lateral, and 3.5 mm ventral) were based on the atlas of Paxinos and Watson (2006). The chronic 22-gauge guide cannula was fixed by dental cement, anchored by four sterile screws (Plastic One Inc., Roanoke, VA, USA), and the wound was sutured. After the surgery, animals were housed individually and allowed to recover for 5 days. During this time, rats were handled daily to adapt to the icv injection procedure (light hand restraint for 1 min). For the icv injection a 28-gauge cannula was connected to a 25 μl Hamilton syringe by a PE-50 tube (BD intramedic polyethylene tubing, Clay Adams, NJ, USA) and a volume of 5 μl was injected over a period of 1 min into the conscious rat.

The correct placement of the cannula was verified after the experiments by injecting 5 μl of 0.1% toluidine blue and visualizing the spreading of the dye throughout the brain ventricular system. No animals had to be excluded due to erroneous placement of the cannula.

### Automated food intake monitoring

The BioDAQ episodic food intake monitoring system (BioDAQ, Research Diets Inc.) was used to investigate the food intake microstructure in rats. This system has been established before for the use in mice (Goebel et al., 2011) and recently also rats (Teuffel et al., 2015).

Rats were habituated for 1 week to the single housing (in regular housing cages with normal bedding and enrichment) and feeding from the hopper and as shown before quickly adapted to these conditions within 2–3 days indicated by normal food intake and regular body weight gain (Teuffel et al., 2015). Water was provided *ad libitum* from regular water bottles. Food was provided in low spill food hoppers placed on a balance. The “bridging phenomenon,” that occurs when retained food spillage underneath the gate causes erroneous measurements, was observed very rarely by use of the diet described above and daily maintenance (cleaning) of the hoppers.

The BioDAQ food intake monitoring system weighs the hopper with food (±0.01 g) every second and detects “not eating” as weight stable and “eating” as weight unstable. “Feeding bouts,” defined as change in stable weight before and after an event, are recorded as vectors with starting time, duration and amount of food consumed (Teuffel et al., 2015). The feeding bouts are separated by an inter-bout interval (IBI) and meals can consist of one or more bouts. Furthermore, meals are separated by inter-meal intervals (IMI), in rats defined by a duration of 15 min (Teuffel et al., 2015). The minimum meal size in rats was defined as 0.01 g and therefore food intake was considered as one meal when the feeding bouts occurred within 15 min of the previous response and the content of food was equal to or greater than 0.01 g (Teuffel et al., 2015). If the interval between meals was greater than 15 min, the feeding bouts were considered as a new meal. Meal parameters assessed in this study encompassed bout size (g/bout), meal size (g/meal), bout frequency (number/period), meal frequency (number/period), inter-meal interval (min), meal duration (min/meal), eating rate (mg/min), time spent in meals (%) as well as the satiety ratio (min/g food eaten) calculated using the parameters described above. These parameters were extracted from the software and visualized using the Data Viewer (BioDAQ Monitoring Software 2.3.07, Research Diets Inc.). Data analysis was performed in Excel (Microsoft). For better comparability between rats fed control or high fat diet, food intake was expressed in kcal/300 g body weight (bw).

## Experimental protocols

### Food intake experiments

In the first experiment icv cannulated rats fed *ad libitum* with standard rodent diet and accustomed to the food intake monitoring system as described above were icv injected with nesfatin-1_30−59_ (0.1, 0.3, or 0.9 nmol/rat) or vehicle (5 μl ddH_2_O) directly before the onset of the dark phase. The dose of nesfatin-1_30−59_ was based on our previous study in mice (Stengel et al., 2012a). The food intake microstructure was assessed over a period of 24 h. The experiment was repeated once in a crossover design; rats were allowed to recover for 5 days in between the experiments. During two experiments, behavior was determined by observation of the locomotor activity and grooming (including washing and licking).

In order to assess whether the effect of nesfatin-1_30−59_ is also mediated peripherally, naïve rats accustomed to the food intake monitoring system, handled for ip injections and fed *ad libitum* with standard rodent diet were injected ip with nesfatin-1_30−59_ (8.1, 24.3, or 72.9 nmol/kg) or vehicle (300 μl saline) directly before the dark phase started. These doses were based on the observation that higher doses are required peripherally compared to brain injections (Shimizu et al., 2009). Therefore, up to 30-times higher doses were used peripherally compared to our icv experiments. The microstructure was assessed over a period of 24 h. This experiment was repeated once in a crossover design. Rats were allowed to recover for 5 days in between the experiments.

Since the hypothalamic regulation of food intake is altered under conditions of DIO (Velloso and Schwartz, 2011), we also investigated the effect of nesfatin-1_30−59_ on the food intake microstructure in chronically icv cannulated DIO rats accustomed to the food intake monitoring system and fed a high fat diet. Nesfatin-1_30−59_ (0.9 nmol/rat, dose based on first experiment of the present study) or vehicle (5 μl ddH_2_O) were injected icv directly at the beginning of the dark phase and the food intake microstructure was assessed over a period of 24 h. The experiment was repeated once in a crossover design and rats were allowed to recover for 5 days in between the experiments.

### Statistical analysis

Distribution of the data was determined by using the Kolmogorov-Smirnov test. Data are expressed as mean ± sem and were analyzed by One-way analysis of variance (ANOVA) followed by Tukey *post hoc* test or Two-way or Three-way ANOVA followed by the Holm–Sidak method. Differences between groups were considered significant when *p* < 0.05 (SigmaStat 3.1., Systat Software, San Jose, CA, USA).

## Results

### Nesfatin-1_30−59_ injected intracerebroventricularly at the onset of the dark phase reduces food intake by decreasing meal size in normal weight rats

**Figure 1:**
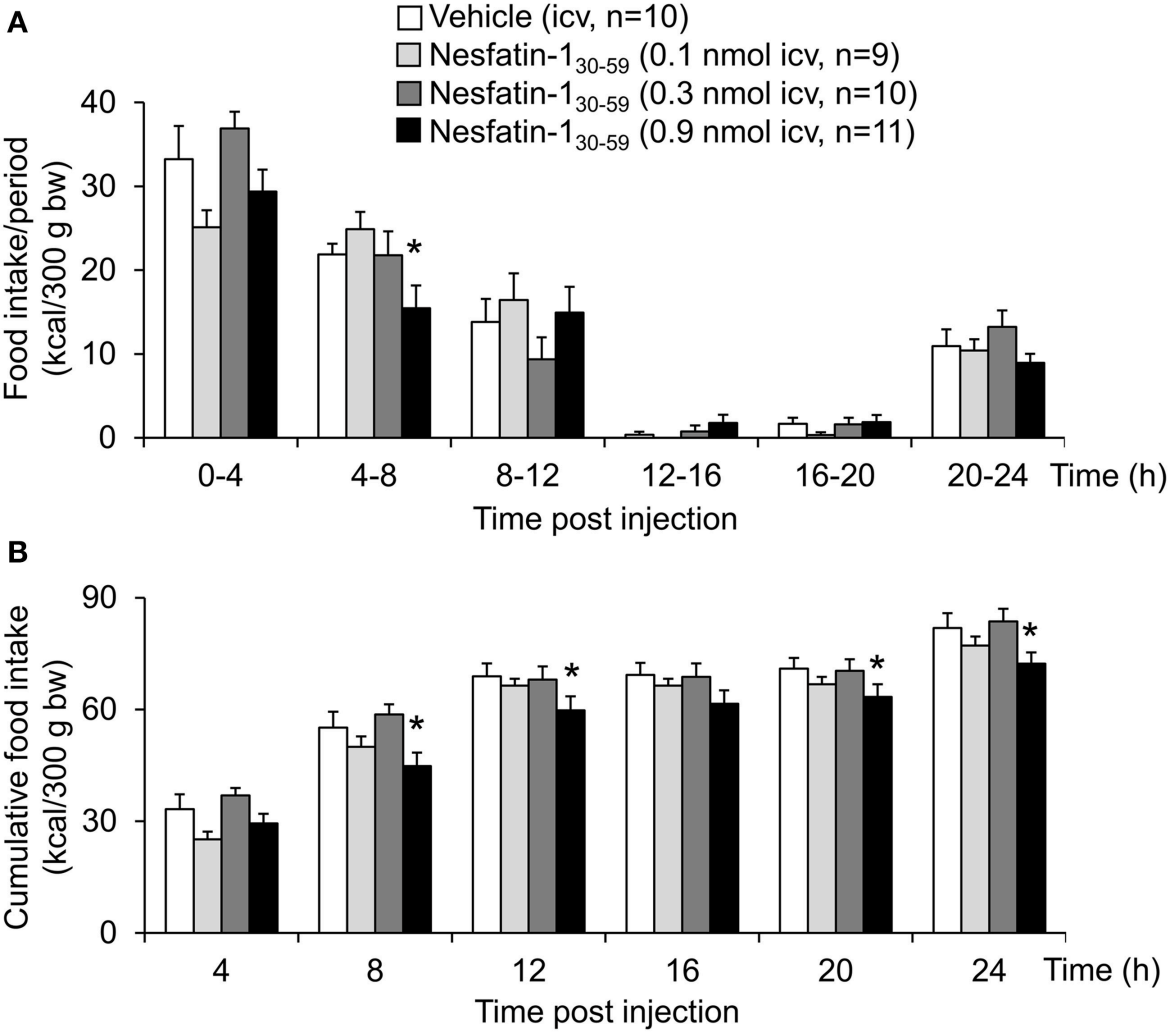
**Nesfatin-1_30−59_ reduces food intake for 24 h in ***ad libitum*** fed normal weight rats**. Nesfatin-1_30−59_ injected intracerebroventricularly (0.1, 0.3, and 0.9 nmol/rat) decreases dark phase food intake of standard rodent diet between the fourth and eighth hour and reduces cumulative food intake for 24 h post injection at a dose of 0.9 nmol/rat. Food intake was measured by an automated episodic food intake monitoring system and expressed as food intake (kcal/300 g bw)/4 h periods **(A)** and cumulative food intake over a period of 24 h **(B)**. Each bar represents the mean ± sem. ^*^p < 0.05 vs. vehicle.

Nesfatin-1_30−59_ (0.1, 0.3, or 0.9 nmol/rat) injected icv in normal weight rats fed *ad libitum* with standard rodent diet induced a dose-related reduction of dark phase food intake with a delayed onset between the 4–8 h period post injection and observed at a dose of 0.9 nmol/rat compared to vehicle (5 μl ddH_2_O, −29%, *p* < 0.05; Figure 1A). Therefore, this dose was used for all further analyses. This reduction resulted in a decrease of cumulative food intake which was observed over the 24-h measurement period (−12% vs. vehicle, *p* < 0.05; Figure 1B) indicating a long-lasting effect. Two-way ANOVA showed a significant influence of dose [*F*_(3, 239)_ = 11.2, *p* < 0.001] and time [*F*_(5, 239)_ = 109.2, *p* < 0.001].

**Figure 2:**
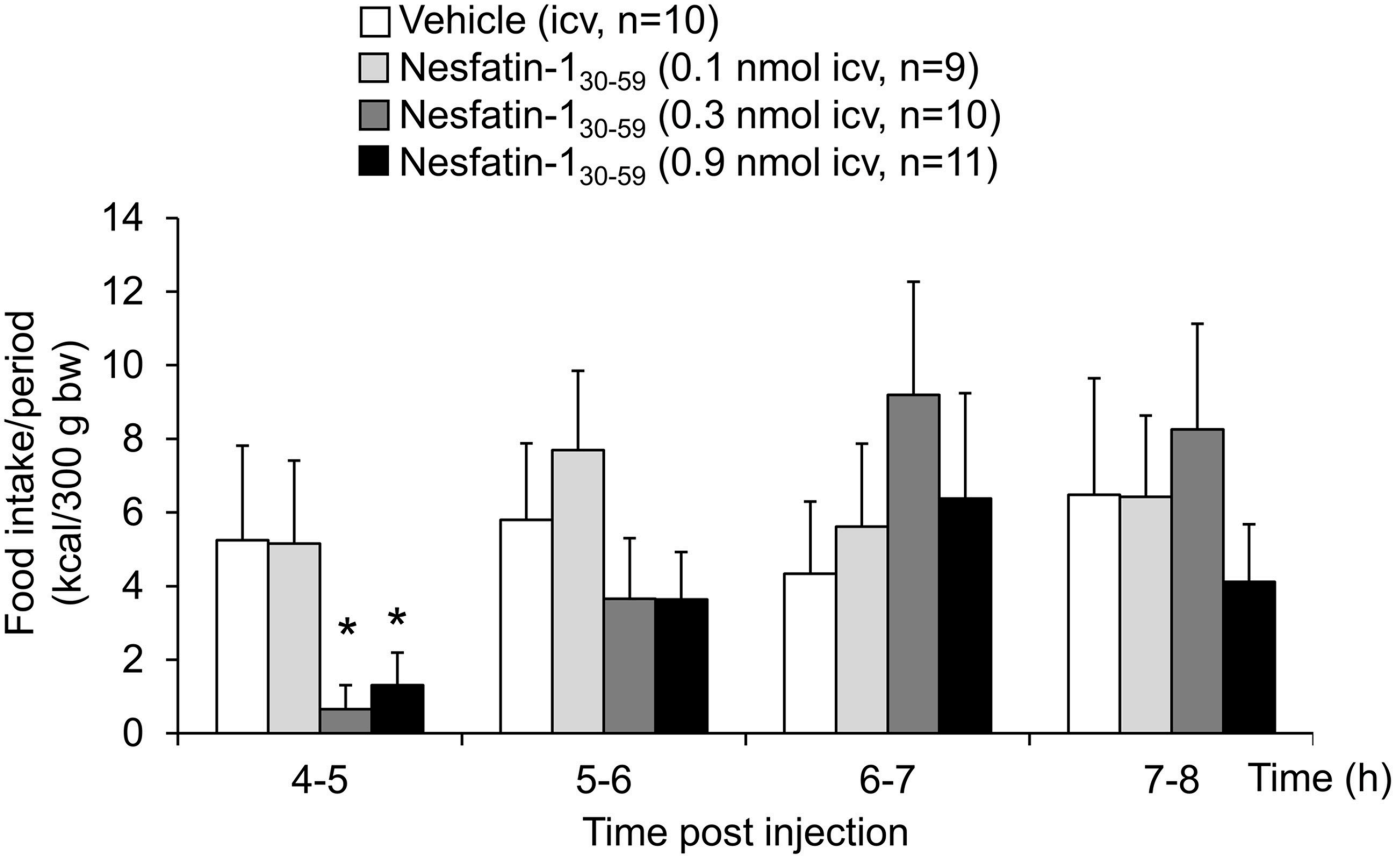
**Nesfatin-1_30−59_ reduces food intake during the fifth hour post injection in normal weight rats**. In rats fed *ad libitum* with standard rat diet, nesfatin-1_30−59_ injected intracerebroventricularly (0.1, 0.3, and 0.9 nmol/rat) induces a dose-related decrease in dark phase food intake during the fifth hour post injection reaching significance at 0.3 and 0.9 nmol/rat. Food intake is expressed as hourly food intake (kcal/300 g bw) during the 4–8 h period post injection. Each bar represents the mean ± sem. ^*^p < 0.05 vs. vehicle.

**Figure 3:**
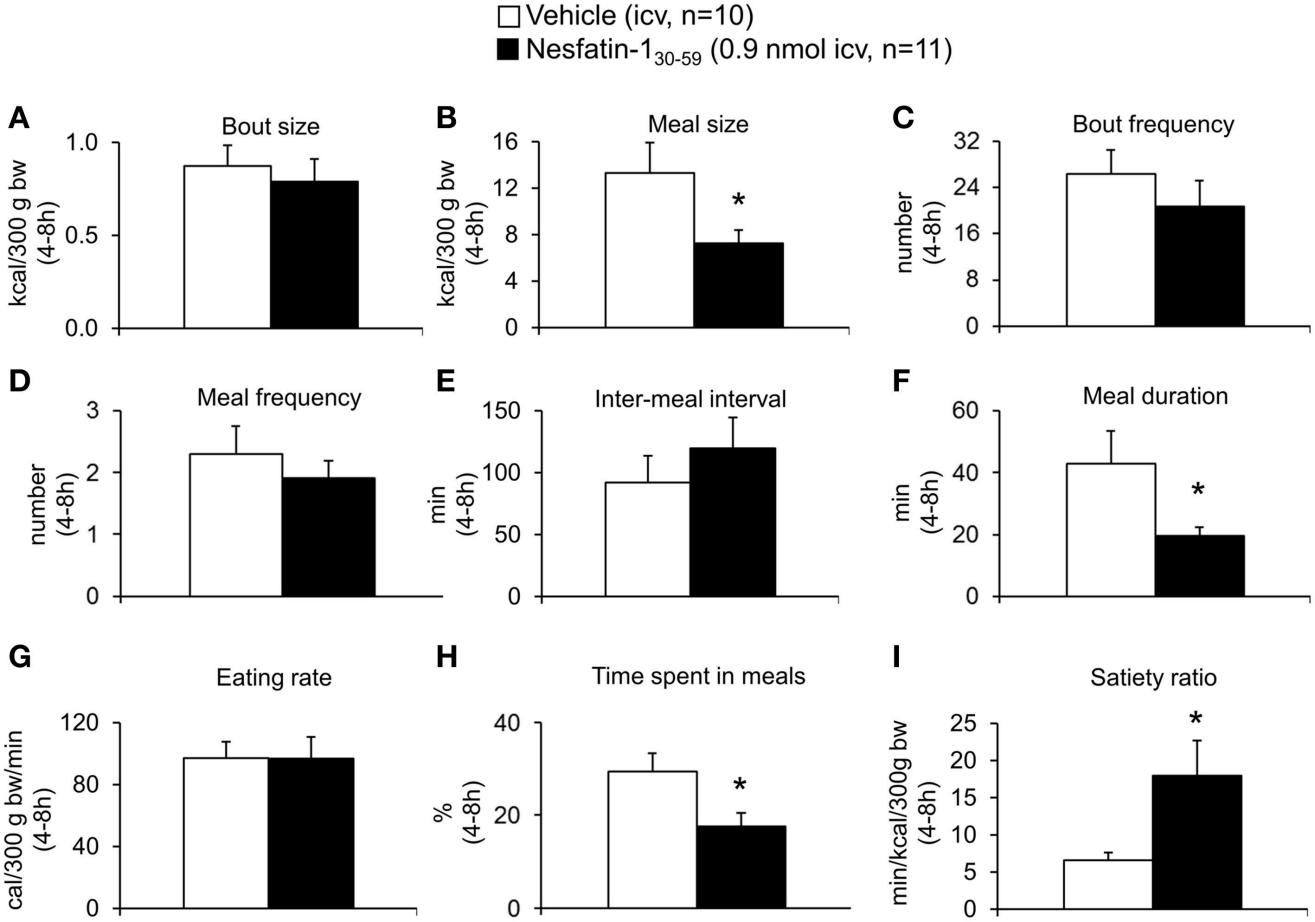
**Nesfatin-1_30−59_ reduces meal size, while meal frequency is not altered in normal weight rats**. Nesfatin-1_30−59_ (0.1, 0.3, and 0.9 nmol/rat) or vehicle (5 μl ddH_2_O) was injected intracerebroventricularly and the food intake microstructure assessed using an automated episodic food intake monitoring system. Due to the strongest reduction in food intake, the dose of 0.9 nmol/rat was used for all further analyses. During the period of 4–8 h post injection, nesfatin-1_30−59_ reduced meal size **(B)**, meal duration **(F)** and the time spent in meals **(H)**, while bout size **(A)**, bout frequency **(C)**, meal frequency **(D)**, inter-meal intervals **(E)** and eating rate **(G)** were not altered compared to vehicle. The satiety ratio was increased following nesfatin-1_30−59_ **(I)**. Each bar represents the mean ± sem. ^*^p < 0.05 vs. vehicle.

Analysis of hourly food intake indicated that the main anorexigenic effect occurred during the fifth hour post injection with a −75% reduction of food intake following nesfatin-1_30−59_ compared to vehicle (*p* = 0.05; Figure 2). Moreover, parameters of the first meal were mostly unaffected by icv nesfatin-1_30−59_ (Table 1) further pointing toward a delayed onset of the anorexigenic action. However, the interval following the first meal was prolonged by nesfatin-1_30−59_ compared to vehicle (+48%, *p* < 0.05; Table 1). Due to the significant reduction of food intake during the 4–8 h period post injection, the underlying food intake microstructure was analyzed during this period. Nesfatin-1_30−59_ (0.9 nmol/rat icv) reduced meal size (−45%, *p* < 0.05; Figure 3B), meal duration (−54%, *p* < 0.05; Figure 3F), and the time spent in meals (−41%, *p* < 0.05; Figure 3H), while bout size (Figure 3A), bout frequency (Figure 3C), meal frequency (Figure 3D), inter-meal intervals (Figure 3E), and eating rate (Figure 3G) were not significantly affected compared to vehicle (*p*>0.05). At the same time, the satiety ratio was increased following nesfatin-1_30−59_ compared to vehicle injected icv (+174%, *p* < 0.05; Figure 3I). Manual observation indicated no abnormal behavior following icv injection of nesfatin-1_30−59_ (data not shown).

**Table 1:** **Nesfatin-1_30−59_ injected icv before the dark phase does not alter the parameters of the first meal in ***ad libitum*** fed normal weight rats**.

**Parameter**	**Group**	
	**Vehicle (5 μl ddH_2_O icv)**	**Nesfatin-1_30−59_ (0.9 nmol icv)**
Latency to first meal (min)	2.09±0.55	1.88±0.58
Size of first meal (kcal/300 g bw)	5.85±0.84	7.73±0.90
Duration of first meal (min)	10.09±1.64	13.54±1.68
Eating rate of first meal (cal/300 g bw/min)	35.80±3.94	29.69±2.86
Inter-meal interval (min)	46.71±6.44	69.32±4.59^*^
Satiety ratio after first meal (min/kcal/300 g bw food eaten)	9.16±1.53	11.80±1.93

icv, intracerebroventricular; mean ± sem; n = 10–11/group;

* *p < 0.05*.

### Nesfatin-1_30−59_ injected intraperitoneally at the onset of the dark phase does not reduce food intake or alter the food intake microstructure of normal weight rats

Nesfatin-1_30−59_ (8.1, 24.3, or 72.9 nmol/kg, ip) injected at higher doses at the onset of the dark phase did not robustly reduce food intake either expressed as food intake/period or cumulative food intake compared to vehicle (*p*>0.05; Table 2). Only the highest dose (72.9 nmol/kg, ip) led to a slight and short-lasting reduction of food intake during the seventh hour post injection compared to vehicle (*p* < 0.05) that did not translate into a reduction of cumulative food intake (*p*>0.05; Table 2). Also analysis of the food intake microstructure in the 4–8 h period (the period when nesfatin-1_30−59_ injected icv exerted its main effects) indicated no alterations of microstructural parameters compared to vehicle injected ip (*p*>0.05; Table 3). Lastly, parameters of the first meal were not affected by nesfatin-1_30−59_ injected ip compared to control (*p*>0.05; Table 4).

**Table 2:** **Nesfatin-1_30−59_ injected ip before the dark phase does not alter food intake in ***ad libitum*** fed normal weight rats**.

**Period**	**Group**			
	**Vehicle (300 μl saline ip)**	**Nesfatin-1_30−59_ (8.1 nmol/kg ip)**	**Nesfatin-1_30−59_ (24.3 nmol/kg ip)**	**Nesfatin-1_30−59_ (72.9 nmol/kg ip)**
**FOOD INTAKE PER PERIOD (KCAL/300 G BW)**				
0–4 h	41.15±2.20	44.00±2.47	44.42±2.21	44.48±2.22
4–8 h	27.70±2.09	22.06±2.54	25.80±2.24	27.39±1.78
8–12 h	13.15±2.03	19.61±2.10	15.56±2.47	17.33±2.24
12–16 h	0.47±0.32	0.63±0.45	1.59±1.06	2.85±1.48
16–20 h	1.21±0.78	0.32±0.28	1.31±0.90	0.78±0.71
20–24 h	12.51±1.28	10.48±0.81	12.54±1.78	10.83±0.91
**CUMULATIVE FOOD INTAKE (KCAL/300 G BW)**				
0–4 h	41.15±2.20	44.00±2.47	44.42±2.21	44.48±2.22
0–8 h	68.84±2.56	66.05±2.30	70.23±2.33	71.86±3.16
0–12 h	81.99±2.76	85.67±2.61	85.79±2.74	89.19±3.36
0–16 h	82.47±2.69	86.29±2.68	87.38±2.44	92.05±3.32
0–20 h	83.67±2.79	86.62±2.64	88.69±2.35	92.82±3.45
0–24 h	96.18±2.91	97.09±2.57	101.23±2.36	103.65±3.30

*ip, intraperitoneal; mean ± sem; n = 14/group; p > 0.05*.

**Table 3:** **Nesfatin-1_30−59_ injected ip before the dark phase does not alter the food intake microstructure in the 4–8 h period post injection in ***ad libitum*** fed normal weight rats**.

**Parameter**	**Group**			
	**Vehicle (300 μl saline ip)**	**Nesfatin-1_30−59_ (8.1 nmol/kg ip)**	**Nesfatin-1_30−59_ (24.3 nmol/kg ip)**	**Nesfatin-1_30−59_ (72.9 nmol/kg ip)**
Food intake 4–5 h (kcal/300 g bw)	5.96±2.13	6.09±2.15	5.26±2.06	8.35±2.87
Food intake 5–6 h (kcal/300 g bw)	3.22±2.09	4.21±1.98	6.95±2.52	12.20±3.31
Food intake 6–7 h (kcal/300 g bw)	14.18±3.34	5.76±1.97	6.63±2.89	4.29±1.99^*^
Food intake 7–8 h (kcal/300 g bw)	4.34±2.00	6.00±2.54	6.97±2.25	2.53±1.50
Bout size 4–8 h (kcal/300 g bw)	0.86±0.06	0.82±0.07	0.87±0.04	0.85±0.05
Meal size 4–8 h (kcal/300 g bw)	17.01±2.39	14.32±1.58	15.35±2.25	18.55±2.11
Bout frequency 4–8 h (number)	32.07±3.39	27.29±1.99	31.00±2.86	32.64±2.83
Meal frequency 4–8 h (number)	1.93±0.22	1.71±0.27	2.00±0.23	1.71±0.19
Inter-meal interval 4–8 h (min)	143.61±31.10	101.41±18.08	147.27±27.32	159.46±31.92
Meal duration 4–8 h (min)	53.67±8.20	47.36±7.52	48.12±8.75	57.40±7.42
Eating rate 4–8 h (cal/300 g bw/min)	109.27±10.94	123.77±11.05	107.61±11.40	125.47±18.96
Time spent in meals 4–8 h (%)	36.65±3.17	29.40±3.94	31.89±3.01	35.49±3.43
Satiety ratio 4–8 h (min/kcal/300 g bw food eaten)	8.17±1.08	7.73±1.66	10.33±1.41	8.47±10.4

ip, intraperitoneal; mean ± sem; n = 14/group;

* *p < 0.05 vs vehicle*.

**Table 4:** **Nesfatin-1_30−59_ injected ip before the dark phase does not alter parameters of the first meal in ***ad libitum*** fed normal weight rats**.

**Parameter**	**Group**			
	**Vehicle (300 μl saline ip)**	**Nesfatin-1_30−59_ (8.1 nmol/kg ip)**	**Nesfatin-1_30−59_ (24.3 nmol/kg ip)**	**Nesfatin-1_30−59_ (72.9 nmol/kg ip)**
Latency to first meal (min)	4.83±2.24	9.23±3.67	4.74±1.84	7.40±5.39
Size of first meal (kcal/300 g bw)	11.08±1.59	7.70±0.85	8.55±1.26	14.00±2.92
Duration of first meal (min)	21.15±3.84	16.95±3.24	17.70±4.17	29.35±8.89
Eating rate of first meal (cal/300 g bw/min)	156.85±12.37	152.62±17.56	134.24±13.45	176.78±17.44
Inter-meal interval (min)	48.17±6.94	36.54±4.90	43.12±3.20	49.28±7.17
Satiety ratio after first meal (min/kcal/300 g bw food eaten)	5.04±0.57	5.72±1.19	9.08±3.49	4.40±0.57

*ip, intraperitoneal; mean ± sem; n = 14/group; p > 0.05*.

### Nesfatin-1_30−59_ injected intracerebroventricularly at the onset of the dark phase reduces food intake by decreasing meal frequency of diet-induced-obese rats fed a high fat diet

**Figure 4:**
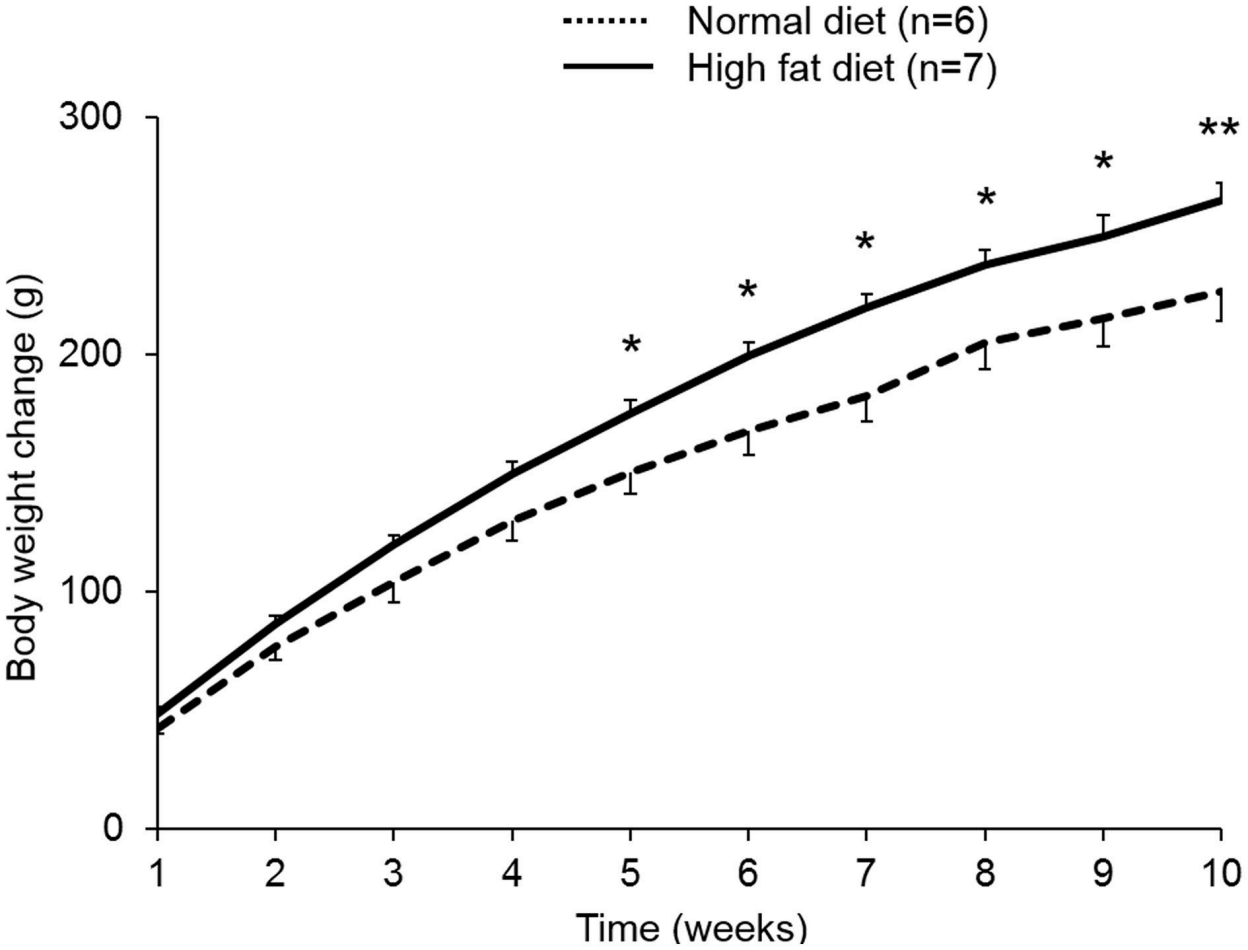
**Rats fed a high fat diet develop diet-induced obesity**. Rats were ***ad libitum*** fed either a high fat diet (45% calories from fat, 35% from carbohydrates, and 20% from protein, 4.7 kcal/g) or standard rodent diet (10% calories from fat, 70% from carbohydrates, and 20% from protein, 3.9 kcal/g) over a period of 10 weeks. Rats that gained most body weight (~50%) were selected for the following experiments as diet-induced obese (DIO) rats. DIO rats showed greater body weight gain compared to rats fed standard rodent diet. Data are expressed as mean ± sem. ^*^p < 0.05 and ^**^p < 0.01 vs. vehicle.

Rats fed a high fat diet (45% calories from fat) for a period of 10 weeks developed DIO and gained significantly more body weight compared to rats fed standard rodent diet (10% calories from fat; +21% at 10 weeks, *p* < 0.01; Figure 4).

**Figure 5:**
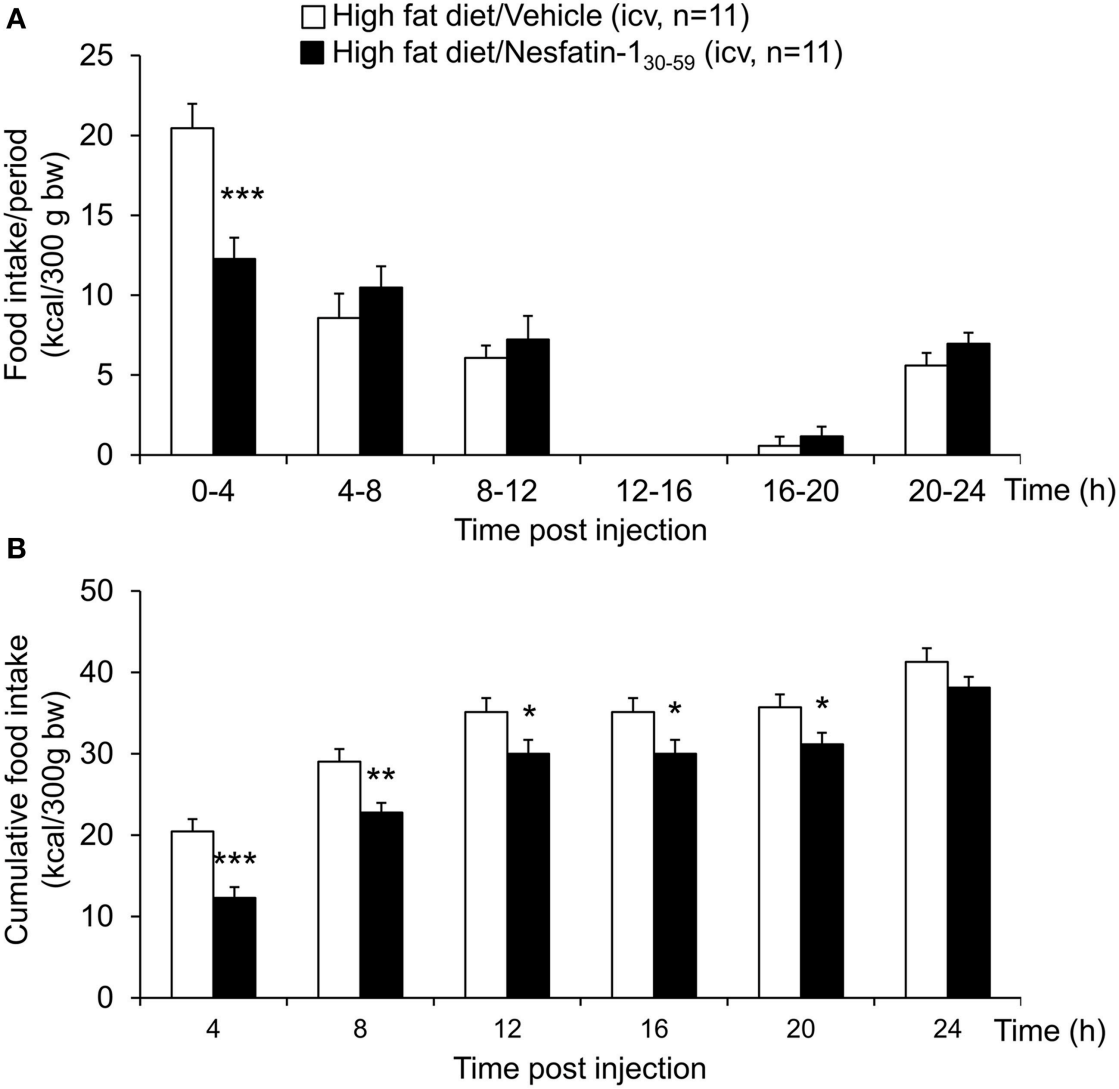
**Nesfatin-1_30−59_ reduces food intake for 8 h in ***ad libitum*** fed diet-induced obese rats**. Nesfatin-1_30−59_ (0.9 nmol/rat) injected intracerebroventricularly decreased dark phase food intake in the first 4 h period post injection, an effect that lasted for 8 h compared to vehicle (5 μl dd H_2_O). Food intake was assessed using an automated episodic food intake monitoring system and expressed as food intake (kcal/300 g bw)/4 h periods **(A)** and cumulative food intake over a period of 24 h **(B)**. Each bar represents the mean ± sem. ^*^p < 0.05 vs. vehicle; ^**^p < 0.01 vs. vehicle; ^***^p < 0.001 vs. vehicle.

**Figure 6:**
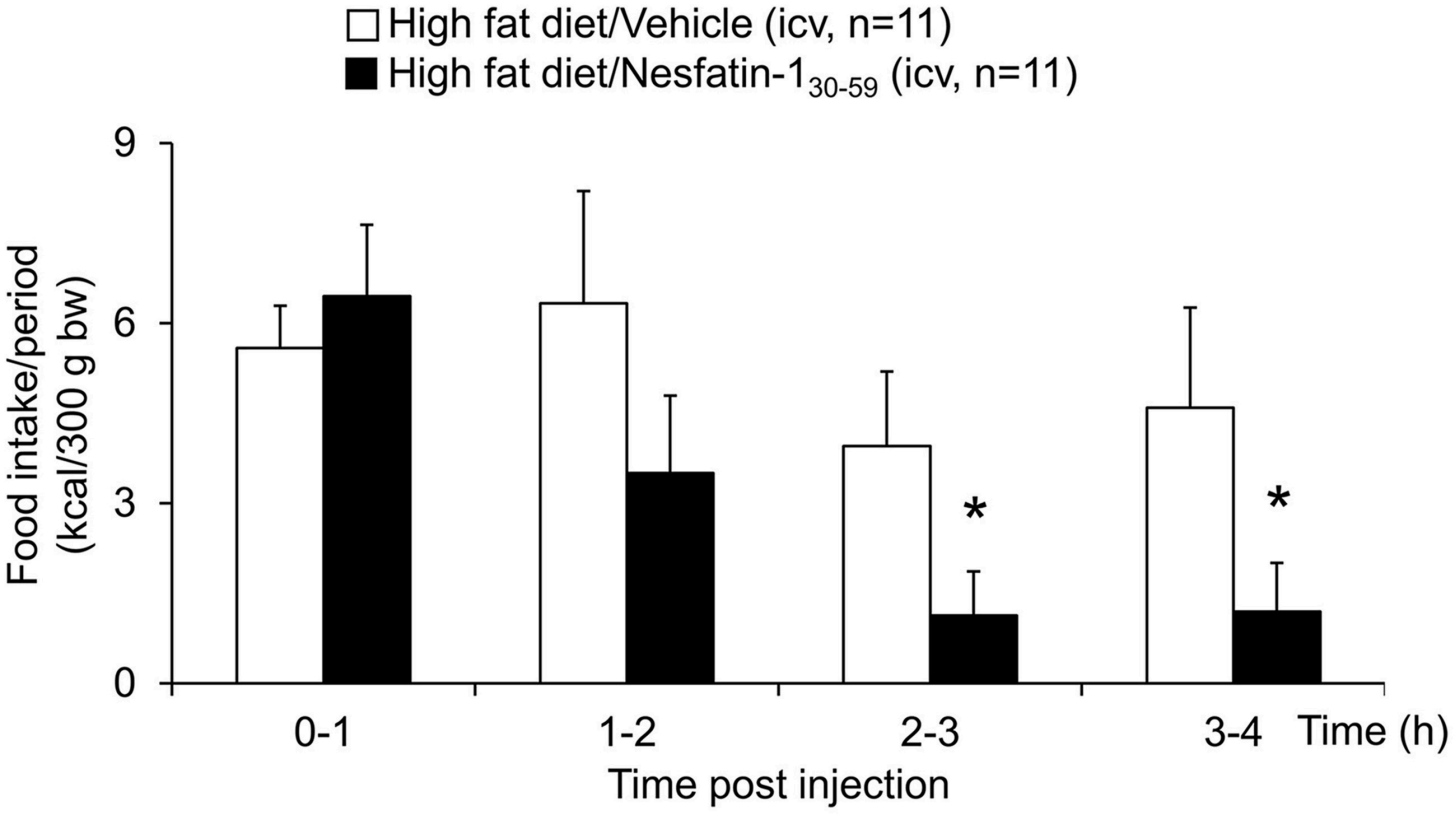
**Nesfatin-1_30−59_ reduces food intake in the third and fourth hour post icv injection in diet-induced obese rats**. Nesfatin-1_30−59_ injected intracerebroventricularly (0.9 nmol/rat) decreased dark phase food intake in *ad libitum* fed rats during the third and fourth hour post injection compared to vehicle (5 μl dd H_2_O). Food intake was assessed using an automated episodic food intake monitoring system and expressed as food intake (kcal/300 g bw)/1 h periods. Each bar represents the mean ± sem. ^*^p < 0.05 vs. vehicle.

When injected icv in DIO rats, nesfatin-1_30−59_ (0.9 nmol/rat) induced a reduction of food intake during the 0–4 h period post injection compared to vehicle (5 μl ddH_2_O, −40%, *p* < 0.001; Figure 5A). This reduction resulted in a decrease of cumulative food intake over a period of 20 h (−13% vs. vehicle, *p* < 0.05; Figure 5B). Two-way ANOVA indicated a significant influence of treatment [*F*_(1, 131)_ = 36.3, *p* < 0.001] and time [*F*_(5, 131)_ = 53.4, *p* < 0.001]. Analysis of hourly food intake during the 0–4 h period showed that the main anorexigenic effect of nesfatin-1_30−59_ occurred during the third and fourth hour post injection (−71 and −74%, respectively, compared to vehicle, *p* < 0.05; Figure 6) pointing toward a delayed effect. In line with this assumption, parameters of the first meal were not significantly altered following nesfatin-1_30−59_ compared to vehicle (Table 5).

**Table 5:** **Nesfatin-1_30−59_ injected icv before the dark phase does not alter parameters of the first meal in diet-induced obese rats fed ***ad libitum*** with high fat diet**.

**Parameter**	**Group**	
	**Vehicle (5 μl ddH_2_O icv)**	**Nesfatin-1_30−59_ (0.9 nmol icv)**
Latency to first meal (min)	14.74±6.05	16.62±9.99
Size of first meal (kcal/300 g bw)	5.98±0.46	6.36±0.89
Duration of first meal (min)	12.96±3.11	13.54±2.85
Eating rate of first meal (cal/300 g bw/min)	71.91±8.76	60.26±12.93
Inter-meal interval (min)	80.66±9.92	128.10±24.60
Satiety ratio after first meal (min/kcal/300 g bw food eaten)	13.76±1.77	22.50±5.00

*icv, intracerebroventricular; mean ± sem; n = 11/group; p > 0.05*.

**Figure 7:**
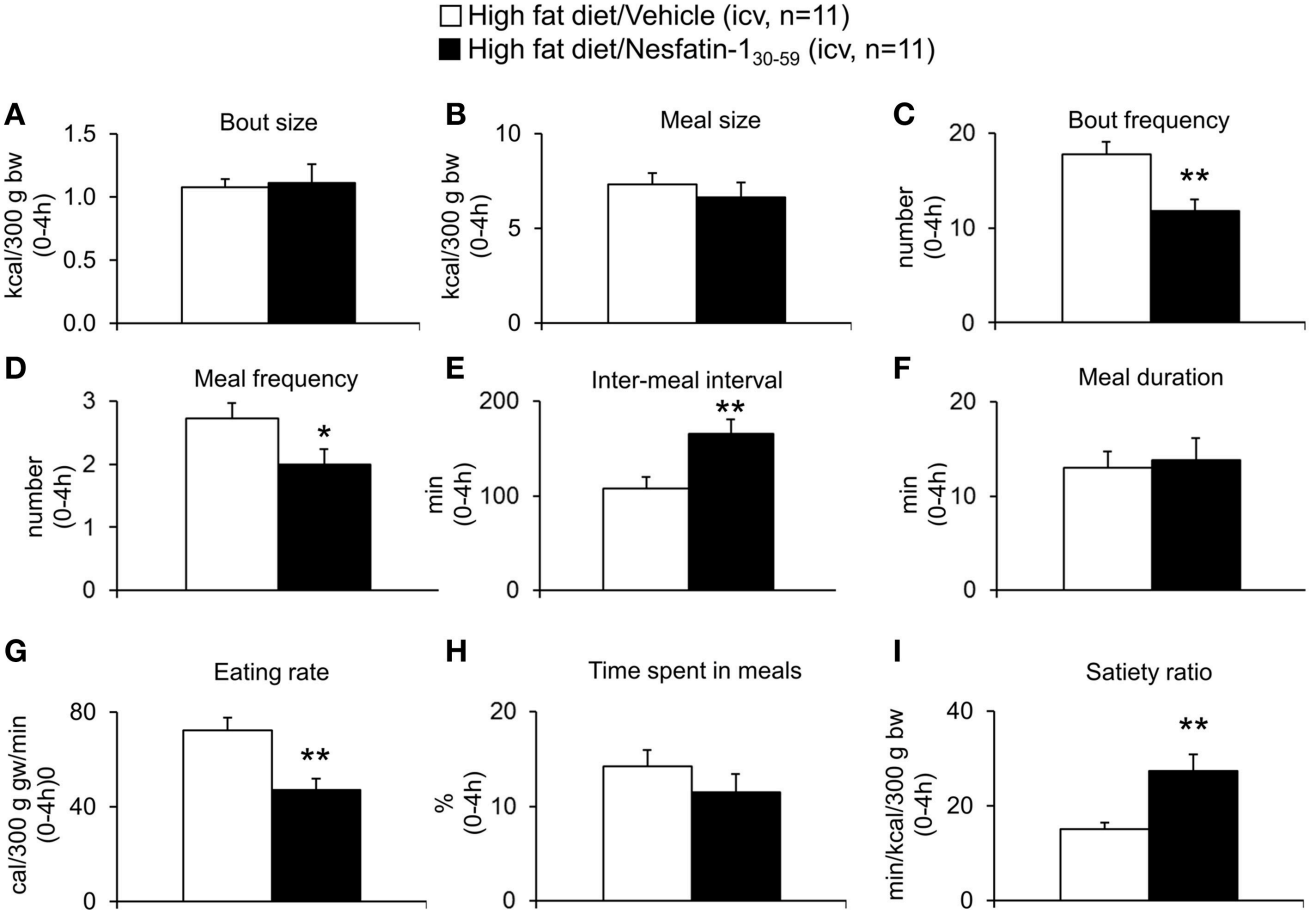
**Nesfatin-1_30−59_ decreases meal frequency, while meal size is not altered in diet-induced obese rats**. DIO rats fed high fat diet were injected intracerebroventricularly with nesfatin-1_30−59_ (0.9 nmol/rat) or vehicle (5 μl dd H_2_O) and the food intake microstructure assessed using an automated episodic food intake monitoring system. During the period of 0–4 h post injection, nesfatin-1_30−59_ reduced bout frequency **(C)**, meal frequency **(D)**, and eating rate **(G)**, while bout size **(A)**, meal size **(B)**, meal duration **(F)**, and the time spent in meals **(H)** were not altered compared to vehicle. Inter-meal intervals **(E)** and the satiety ratio **(I)** were increased following nesfatin-1_30−59_. Each bar represents the mean ± sem. ^*^p < 0.05 and ^**^p < 0.01 vs. vehicle.

Based on the finding that nesfatin-1_30−59_ already decreased food intake in DIO rats during the 0–4 h period post injection, the microstructure was analyzed during this period. Nesfatin-1_30−59_ (0.9 nmol/rat, icv) reduced bout frequency (−33%, *p* < 0.05; Figure 7C), meal frequency (−27%, *p* < 0.05; Figure 7D), and eating rate (−35%, *p* < 0.01; Figure 7G), while bout size (Figure 7A), meal size (Figure 7B), meal duration (Figure 7F), and the time spent in meals (Figure 7H) were not significantly affected compared to vehicle (*p*>0.05). Moreover, inter-meal intervals (+53%, Figure 7E) and the satiety ratio (+81%, Figure 7I) were increased following icv injection of nesfatin-1_30−59_ (*p* < 0.01 vs. vehicle).

The combined analysis of food intake data of rats fed control or high fat diet using Three-way ANOVA indicated a significant influence of diet [*F*_(1, 257)_ = 83.5, *p* < 0.001], time [*F*_(5, 257)_ = 108.1, *p* < 0.001], and interaction of diet and time [*F*_(5, 257)_ = 10.6, *p* < 0.001] as well as an interaction of treatment and time [*F*_(5, 257)_ = 2.6, *p* < 0.05].

## Discussion

In the present study we investigated the effect of nesfatin-1_30−59_ on feeding behavior in rats under normal weight and diet-induced obese conditions. Nesfatin-1_30−59_ injected icv reduced dark phase food intake in normal weight rats fed a standard rodent diet. This effect was delayed in onset as parameters of the first meal were not altered with a maximum reduction of food intake observed during the fifth hour post injection and long lasting as it resulted in a reduction of cumulative food intake over the 24 h observation period. These data are in accordance with a previous study in rats where the main reduction following icv full length nesfatin-1_1−82_ was observed during the third hour post injection and also observed over the whole measurement period of 6 h (Stengel et al., 2009a). The difference in the onset of action (third hour after nesfatin-1_1−82_ vs. fifth hour after nesfatin-1_30−59_) may be due to differential diffusion capacities following icv injection and—more importantly—differential interaction with the yet unknown nesfatin-1 receptor. Lastly, also the interaction with (an) additional receptor(s) has to be considered.

Interestingly, higher doses of nesfatin-1_30−59_ were needed to induce an anorexigenic effect. While nesfatin-1_1−82_ injected icv reduced food intake in rats following injection of 5 pmol, nesfatin-1_30−59_ exerted an anorexigenic effect after icv injection of 0.9 nmol/rat. This difference is likely to result from differential ligand-receptor interaction with full length nesfatin-1_1−82_ being more potent to induce the food intake inhibitory effect at low doses. Whether the difference in potency is due to more selective binding of nesfatin-1_1−82_ to the receptor, slower dissociation or a more potent stimulation of the receptor compared to nesfatin-1_30−59_ will have to be investigated in future studies. The effective dose observed here in rats is comparable to the anorexigenic doses described before in mice (0.3 and 0.9 nmol/mouse nesfatin-1_30−59_ icv; Stengel et al., 2012a).

After establishing nesfatin-1_30−59_ also as an active fragment of nesfatin-1_1−82_ to reduce food intake in rats we investigated the underlying food intake microstructure. Nesfatin-1_30−59_ injected icv reduced food intake by reducing meal size, while meal frequency and inter-meal intervals were not altered. This pattern indicates an increase of satiation (earlier termination of a meal), whereas satiety (later initiation of a new meal after one meal is completed) is not affected. This pattern is different from the one observed before for mice where satiety (reduction in meal frequency) was induced while satiation (meal size was not altered) was not affected by icv nesfatin-1_30−59_ (Stengel et al., 2012a). This difference points toward species differences and highlights the need for cautious translation of results from one species to another. Interestingly, in mice full length nesfatin-1_1−82_ injected icv increased both satiation and satiety (Goebel et al., 2011). This different pharmacodynamics is likely due to a differential receptor binding of full length nesfatin-1_1−82_ and its active core, nesfatin-1_30−59_.

Early on, the downstream signaling of NUCB2/nesfatin-1 has been investigated. It has been shown that oxytocin is involved in the mediation of nesfatin-1_1−82_'s anorexigenic effect as nesfatin-1_1−82_ activates oxytocin containing neurons in the paraventricular nucleus of the hypothalamus (PVN) and central administration of the oxytocin-receptor antagonist, H4928 abolished the food intake reduction induced by nesfatin-1_1−82_ in rats (Maejima et al., 2009). Moreover, an involvement of proopiomelanocortin (POMC) and cocaine and amphetamine-regulated transcript (CART) has been indicated as well based on the following observations: First, nesfatin-1_30−59_ was shown to upregulate the mRNA expression of POMC and CART in mice (Shimizu et al., 2009). Second, blockade of α-melanocyte stimulating hormone (α-MSH) signaling, a major anorexigenic cleavage product of POMC (Schwartz et al., 2000), using the melanocortin receptor 3/4 antagonist, SHU9119 also blocked nesfatin-1_1−82_'s anorexigenic effect (Oh-I et al., 2006). Third, brain ventricular injection of the melanocortin receptor 3/4 agonist, MTII decreased food intake by reducing meal size, while no effect on meal frequency was observed (Azzara et al., 2002; Berthoud et al., 2006), resembling the pattern of action observed after icv injection of nesfatin-1_30−59_ in the present study. Since oxytocin increases the release of POMC in the nucleus of the solitary tract (NTS), nesfatin-1_30−59_ is likely to act *via* an oxytocin → POMC → α-MSH/melanocortin receptor 3/4 pathway to inhibit food intake in rats.

Despite the fact that NUCB2/nesfatin-1 is predominantly expressed in the stomach (Stengel et al., 2009b), the role of peripheral NUCB2/nesfatin-1 is far from being clear. While one study reported an anorexigenic effect following ip injection of nesfatin-1_30−59_ in mice (Shimizu et al., 2009), other studies in rats (Stengel et al., 2009a), or mice (Goebel et al., 2011) did not detect any effect. Similarly, in the present study we did not find an anorexigenic effect of nesfatin-1_30−59_ injected ip in normal weight rats although up to 30-fold higher doses were used. This difference may be due to species differences (mice vs. rats) or different doses used (up to ~40 nmol/mouse vs. ~22 nmol/rat in the present study). However, these findings clearly point toward a central mode of action of nesfatin-1 to reduce food intake, while peripheral nesfatin-1 is likely to have a different main effect such as glucose control (Nakata and Yada, 2013).

Signaling of several food intake regulatory hormones is altered under conditions of obesity (Hellström, 2013). These changes encompass a decrease in circulating ghrelin levels (Ariyasu et al., 2002) which may represent a compensatory action to prevent further overeating. On the other side, the postprandial release of anorexigenic signals such as peptide YY (Xu et al., 2011) is blunted under these condition which is likely to result in reduced anorexigenic signaling. Therefore, we investigated the effect of nesfatin-1_30−59_ also in DIO rats fed a high fat diet. The anorexigenic effect of nesfatin-1_30−59_ was retained in DIO rats with a delayed onset (parameters of the first meal were not changed) and a long duration of action. Interestingly, the main food intake inhibitory effect was already observed during the third hour post injection (−71%), while in normal weight rats the main reduction occurred later in the fifth hour post nesfatin-1_30−59_ icv injection (−75%). Moreover, the effect translated into a reduction of food intake over a 20 h period (−13%), while in normal weight rats the effect lasted for 20 h (−12%). Lastly, the underlying food intake microstructure was different in DIO rats compared to normal weight rats with a decrease in meal frequency and an unaltered meal size under conditions of high fat feeding indicating an increase of satiety while satiation is not altered. Taken together, these findings give rise to differential receptor binding, altered diffusion and—most importantly—point toward a differential downstream signaling under conditions of DIO compared to normal weight rats.

Interestingly, it was recently shown that rats fed a high fat diet display significantly lowered plasma NUCB2/nesfatin-1 levels (Haghshenas et al., 2014; Mohan et al., 2014). Although the cellular action on the so far unknown receptor is yet to be established, the chronically reduced circulating NUCB2/nesfatin-1 levels may result in a sensitization of the receptor and lead to a stronger/more rapid response to icv nesfatin-1_30−59_. Early on, it was shown that nesfatin-1_1−82_ acts in a leptin-independent fashion (Oh-I et al., 2006; Maejima et al., 2009). However, the corticotropin releasing factor (CRF) receptor 2, shown to play a role in nesfatin-1_1−82_'s anorexigenic signaling under normal weight conditions (Stengel et al., 2009a), may be important under DIO conditions as well. An earlier study showed that brain injection of the selective CRF_2_ agonist, urocortin 3 (1 μg/rat) reduced food intake with a delayed onset (between 3 and 4 h) by decreasing meal frequency while meal size was not altered in rats (Fekete et al., 2007), a pattern very similar to the one observed following icv nesfatin-1_30−59_ observed under DIO conditions in the present study. The predominance of the CRF_2_ downstream signaling pathway in the mediation of nesfatin-1_30−59_'s anorexigenic action might result from reduced processing of POMC to α-MSH under conditions of obesity due to reduced prohormone convertase two levels (Çakir et al., 2013).

In conclusion, these results provide pharmacological evidence that nesfatin-1_30−59_ is the active core of full length nesfatin-1_1−82_ also in rats that reduces food intake in normal weight rats fed standard rodent diet as well as DIO rats fed a high fat diet. Interestingly, the food intake microstructure differs with an increase of satiation under normal weight and an induction of satiety under DIO conditions. This may result from a downstream nesfatin-1_30−59_ → oxytocin → POMC → α-MSH/melanocortin receptor 3/4 signaling in normal weight rats while in DIO rats the nesfatin-1_30−59_ → CRF/CRF_2_ pathway may predominate. The effect of nesfatin-1_30−59_ is centrally mediated as ip injection of higher doses had no effect. Therefore, the main effect of peripheral nesfatin-1 remains to be established.

### Conflict of interest statement

The authors declare that the research was conducted in the absence of any commercial or financial relationships that could be construed as a potential conflict of interest.

## References

[B1] Al Massadi O. Lear P. V. Müller T. D. Lopez M. Dieguez C. Nogueiras R.. (2014). Review of novel aspects of the regulation of ghrelin secretion. Curr. Drug Metab. 15, 398–413. 10.2174/1389200215666140505153723 24813425

[B2] Ariyasu H. Takaya K. Hosoda H. Iwakura H. Ebihara K. Mori K.. (2002). Delayed short-term secretory regulation of ghrelin in obese animals: evidenced by a specific RIA for the active form of ghrelin. Endocrinology 143, 3341–3350. 10.1210/en.2002-220225 12193546

[B3] Atsuchi K. Asakawa A. Ushikai M. Ataka K. Tsai M. Koyama K.. (2010). Centrally administered nesfatin-1 inhibits feeding behaviour and gastroduodenal motility in mice. Neuroreport 21, 1008–1011. 10.1097/wnr.0b013e32833f7b96 20827224

[B4] Azzara A. V. Sokolnicki J. P. Schwartz G. J. (2002). Central melanocortin receptor agonist reduces spontaneous and scheduled meal size but does not augment duodenal preload-induced feeding inhibition. Physiol. Behav. 77, 411–416. 10.1016/S0031-9384(02)00883-1 12419417

[B5] Berthoud H.-R. Sutton G. M. Townsend R. L. Patterson L. M. Zheng H. (2006). Brainstem mechanisms integrating gut-derived satiety signals and descending forebrain information in the control of meal size. Physiol. Behav. 89, 517–524. 10.1016/j.physbeh.2006.08.018 16996546

[B6] Çakir I. Cyr N. E. Perello M. Litvinov B. P. Romero A. Stuart R. C.. (2013). Obesity induces hypothalamic endoplasmic reticulum stress and impairs proopiomelanocortin (POMC) post-translational processing. J. Biol. Chem. 288, 17675–17688. 10.1074/jbc.M113.475343 23640886 PMC3682568

[B7] Chen X. Dong J. Jiang Z.-Y. (2012). Nesfatin-1 influences the excitability of glucosensing neurons in the hypothalamic nuclei and inhibits the food intake. Regul. Pept. 177, 21–26. 10.1016/j.regpep.2012.04.003 22561448

[B8] Crujeiras A. B. Carreira M. C. Cabia B. Andrade S. Amil M. Casanueva F. F. (2015). Leptin resistance in obesity: an epigenetic landscape. Life Sci. 140, 57–63. 10.1016/j.lfs.2015.05.003 25998029

[B9] Dong J. Guan H.-Z. Jiang Z.-Y. Chen X. (2014). Nesfatin-1 influences the excitability of glucosensing neurons in the dorsal vagal complex and inhibits food intake. PLoS ONE 9:e98967. 10.1371/journal.pone.0098967 24906120 PMC4048226

[B10] Fekete E. M. Inoue K. Zhao Y. Rivier J. E. Vale W. W. Szücs A.. (2007). Delayed satiety-like actions and altered feeding microstructure by a selective type 2 corticotropin-releasing factor agonist in rats: intra-hypothalamic urocortin 3 administration reduces food intake by prolonging the post-meal interval. Neuropsychopharmacology 32, 1052–1068. 10.1038/sj.npp.1301214 17019404 PMC2748839

[B11] Geary N. (2005). A new way of looking at eating. Am. J. Physiol. Regul. Integr. Comp. Physiol. 288, R1444–R1446. 10.1152/ajpregu.00066.2005 15886354

[B12] Goebel M. Stengel A. Wang L. Taché Y. (2011). Central nesfatin-1 reduces the nocturnal food intake in mice by reducing meal size and increasing inter-meal intervals. Peptides 32, 36–43. 10.1016/j.peptides.2010.09.027 20933030 PMC3010516

[B13] Gonzalez R. Kerbel B. Chun A. Unniappan S. (2010). Molecular, cellular and physiological evidences for the anorexigenic actions of nesfatin-1 in goldfish. PLoS ONE 5:e15201. 10.1371/journal.pone.0015201 21151928 PMC2997068

[B14] Haghshenas R. Jafari M. Ravasi A. Kordi M. Gilani N. Shariatzadeh M.. (2014). The effect of eight weeks endurance training and high-fat diet on appetite-regulating hormones in rat plasma. Iran. J. Basic Med. Sci. 17, 237–243. 24904715 PMC4046239

[B15] Hellström P. M. (2013). Satiety signals and obesity. Curr. Opin. Gastroenterol. 29, 222–227. 10.1097/MOG.0b013e32835d9ff8 23314812

[B16] Kerbel B. Unniappan S. (2012). Nesfatin-1 suppresses energy intake, co-localises ghrelin in the brain and gut, and alters ghrelin, cholecystokinin and orexin mRNA expression in goldfish. J. Neuroendocrinol. 24, 366–377. 10.1111/j.1365-2826.2011.02246.x 22023656

[B17] Könczöl K. Pintér O. Ferenczi S. Varga J. Kovács K. Palkovits M.. (2012). Nesfatin-1 exerts long-term effect on food intake and body temperature. Int. J. Obes. 36, 1514–1521. 10.1038/ijo.2012.2 22290539

[B18] Lutz T. A. Woods S. C. (2012). Overview of animal models of obesity. Curr. Protoc. Pharmacol. Chapter 5, Unit 5.61. 10.1002/0471141755.ph0561s58 22948848 PMC3482633

[B19] Maejima Y. Sedbazar U. Suyama S. Kohno D. Onaka T. Takano E.. (2009). Nesfatin-1-regulated oxytocinergic signaling in the paraventricular nucleus causes anorexia through a leptin-independent melanocortin pathway. Cell Metab. 10, 355–365. 10.1016/j.cmet.2009.09.002 19883614

[B20] Mohan H. Ramesh N. Mortazavi S. Le A. Iwakura H. Unniappan S. (2014). Nutrients differentially regulate nucleobindin-2/nesfatin-1 *in vitro* in cultured stomach ghrelinoma (MGN3-1) cells and *in vivo* in male mice. PLoS ONE 9:e115102. 10.1371/journal.pone.0115102 25506938 PMC4266631

[B21] Nakata M. Yada T. (2013). Role of NUCB2/nesfatin-1 in glucose control: diverse functions in islets, adipocytes and brain. Curr. Pharm. Des. 19, 6960–6965. 10.2174/138161281939131127130112 23537085

[B22] Oh-I S. Shimizu H. Satoh T. Okada S. Adachi S. Inoue K.. (2006). Identification of nesfatin-1 as a satiety molecule in the hypothalamus. Nature 443, 709–712. 10.1038/nature05162 17036007

[B23] Pan W. Hsuchou H. Kastin A. J. (2007). Nesfatin-1 crosses the blood-brain barrier without saturation. Peptides 28, 2223–2228. 10.1016/j.peptides.2007.09.005 17950952

[B24] Paxinos G. Watson C. (2006). The Rat Brain in Stereotaxic Coordinates. London, UK: Academic Press.

[B25] Schwartz M. W. Woods S. C. Porte D. Seeley R. J. Baskin D. G. (2000). Central nervous system control of food intake. Nature 404, 661–671. 10.1038/35007534 10766253

[B26] Shimizu H. Oh-I S. Hashimoto K. Nakata M. Yamamoto S. Yoshida N.. (2009). Peripheral administration of nesfatin-1 reduces food intake in mice: the leptin-independent mechanism. Endocrinology 150, 662–671. 10.1210/en.2008-0598 19176321

[B27] Stengel A. Coskun T. Goebel M. Wang L. Craft L. Alsina-Fernandez J.. (2010a). Central injection of the stable somatostatin analog ODT8-SST induces a somatostatin2 receptor-mediated orexigenic effect: role of neuropeptide Y and opioid signaling pathways in rats. Endocrinology 151, 4224–4235. 10.1210/en.2010-0195 20610566 PMC2940496

[B28] Stengel A. Goebel M. Wang L. Rivier J. Kobelt P. Mönnikes H.. (2009a). Central nesfatin-1 reduces dark-phase food intake and gastric emptying in rats: differential role of corticotropin-releasing factor2 receptor. Endocrinology 150, 4911–4919. 10.1210/en.2009-0578 19797401 PMC2775975

[B29] Stengel A. Goebel M. Wang L. Rivier J. Kobelt P. Mönnikes H.. (2010b). Selective central activation of somatostatin receptor 2 increases food intake, grooming behavior and rectal temperature in rats. J. Physiol. Pharmacol. 61, 399–407. 20814067 PMC4040268

[B30] Stengel A. Goebel M. Yakubov I. Wang L. Witcher D. Coskun T.. (2009b). Identification and characterization of nesfatin-1 immunoreactivity in endocrine cell types of the rat gastric oxyntic mucosa. Endocrinology 150, 232–238. 10.1210/en.2008-0747 18818289 PMC2630900

[B31] Stengel A. Goebel-Stengel M. Wang L. Kato I. Mori M. Taché Y. (2012a). Nesfatin-1(30-59) but not the N- and C-terminal fragments, nesfatin-1(1-29) and nesfatin-1(60-82) injected intracerebroventricularly decreases dark phase food intake by increasing inter-meal intervals in mice. Peptides 35, 143–148. 10.1016/j.peptides.2012.03.015 22682899 PMC3372867

[B32] Stengel A. Hofmann T. Goebel-Stengel M. Lembke V. Ahnis A. Elbelt U.. (2013a). Ghrelin and NUCB2/nesfatin-1 are expressed in the same gastric cell and differentially correlated with body mass index in obese subjects. Histochem. Cell Biol. 139, 909–918. 10.1007/s00418-013-1087-8 23515787

[B33] Stengel A. Mori M. Taché Y. (2013b). The role of nesfatin-1 in the regulation of food intake and body weight: recent developments and future endeavors. Obes. Rev. 14, 859–870. 10.1111/obr.12063 23980879 PMC3810163

[B34] Stengel A. Taché Y. (2012b). Gastric peptides and their regulation of hunger and satiety. Curr. Gastroenterol. Rep. 14, 480–488. 10.1007/s11894-012-0291-3 23001831 PMC3482275

[B35] Strubbe J. H. Woods S. C. (2004). The timing of meals. Psychol. Rev. 111, 128–141. 10.1037/0033-295X.111.1.128 14756590

[B36] Teuffel P. Wang L. Goebel-Stengel M. Prinz P. Kobelt P. Scharner S.. (2015). Treatment with ghrelin-O-acyltransferase (GOAT) inhibitor Go-CoA-Tat reduces food intake by reducing meal frequency in rats. *J. Physiol*. Pharmacol. 66, 493–503. 26348074

[B37] Velloso L. A. Schwartz M. W. (2011). Altered hypothalamic function in diet-induced obesity. Int. J. Obes. 35, 1455–1465. 10.1038/ijo.2011.56 21386802 PMC3383790

[B38] Xia Z.-F. Fritze D. M. Li J.-Y. Chai B. Zhang C. Zhang W.. (2012). Nesfatin-1 inhibits gastric acid secretion via a central vagal mechanism in rats. Am. J. Physiol. Gastrointest. Liver Physiol. 303, G570–G577. 10.1152/ajpgi.00178.2012 22723266 PMC3468549

[B39] Xu J. McNearney T. A. Chen J. D. (2011). Impaired postprandial release/syntheses of ghrelin and PYY(3-36) and blunted responses to exogenous ghrelin and PYY(3-36) in a rodent model of diet-induced obesity. J. Gastroenterol. Hepatol. 26, 700–705. 10.1111/j.1440-1746.2010.06563.x 21054519

[B40] Yosten G. L. C. Samson W. K. (2009). Nesfatin-1 exerts cardiovascular actions in brain: possible interaction with the central melanocortin system. Am. J. Physiol. Regul. Integr. Comp. Physiol. 297, R330–R336. 10.1152/ajpregu.90867.2008 19474390 PMC2724238

[B41] Yosten G. L. C. Samson W. K. (2010). The anorexigenic and hypertensive effects of nesfatin-1 are reversed by pretreatment with an oxytocin receptor antagonist. Am. J. Physiol. Regul. Integr. Comp. Physiol. 298, R1642–R1647. 10.1152/ajpregu.00804.2009 20335376 PMC2886698

